# eHealth Applications to Support Independent Living of Older Persons: Scoping Review of Costs and Benefits Identified in Economic Evaluations

**DOI:** 10.2196/24363

**Published:** 2021-03-09

**Authors:** Sandra Sülz, Hilco J van Elten, Marjan Askari, Anne Marie Weggelaar-Jansen, Robbert Huijsman

**Affiliations:** 1 Erasmus School of Health Policy & Management Rotterdam Netherlands; 2 Clinical Informatics, Eindhoven University of Technology Eindhoven Netherlands; 3 Geriant Heerhugowaard Netherlands

**Keywords:** cost, benefit, eHealth, aged, economic evaluation

## Abstract

**Background:**

eHealth applications are constantly increasing and are frequently considered to constitute a promising strategy for cost containment in health care, particularly if the applications aim to support older persons. Older persons are, however, not the only major eHealth stakeholder. eHealth suppliers, caregivers, funding bodies, and health authorities are also likely to attribute value to eHealth applications, but they can differ in their value attribution because they are affected differently by eHealth costs and benefits. Therefore, any assessment of the value of eHealth applications requires the consideration of multiple stakeholders in a holistic and integrated manner. Such a holistic and reliable value assessment requires a profound understanding of the application’s costs and benefits. The first step in measuring costs and benefits is identifying the relevant costs and benefit categories that the eHealth application affects.

**Objective:**

The aim of this study is to support the conceptual phase of an economic evaluation by providing an overview of the relevant direct and indirect costs and benefits incorporated in economic evaluations so far.

**Methods:**

We conducted a systematic literature search covering papers published until December 2019 by using the Embase, Medline Ovid, Web of Science, and CINAHL EBSCOhost databases. We included papers on eHealth applications with web-based contact possibilities between clients and health care providers (mobile health apps) and applications for self-management, telehomecare, telemedicine, telemonitoring, telerehabilitation, and active healthy aging technologies for older persons. We included studies that focused on any type of economic evaluation, including costs and benefit measures.

**Results:**

We identified 55 papers with economic evaluations. These studies considered a range of different types of costs and benefits. Costs pertained to implementation activities and operational activities related to eHealth applications. Benefits (or consequences) could be categorized according to stakeholder groups, that is, older persons, caregivers, and health care providers. These benefits can further be divided into stakeholder-specific outcomes and resource usage. Some cost and benefit types have received more attention than others. For instance, patient outcomes have been predominantly captured via quality-of-life considerations and various types of physical health status indicators. From the perspective of resource usage, a strong emphasis has been placed on home care visits and hospital usage.

**Conclusions:**

Economic evaluations of eHealth applications are gaining momentum, and studies have shown considerable variation regarding the costs and benefits that they include. We contribute to the body of literature by providing a detailed and up-to-date framework of cost and benefit categories that any interested stakeholder can use as a starting point to conduct an economic evaluation in the context of independent living of older persons.

## Introduction

### Background and Motivation

The use of information and communication technologies in health care is regarded as an important piece of the puzzle of increasing health care costs and demand [1], particularly if it targets older persons with substantial health care costs [2]. Induced by an aging population that stays home longer, an increase in self-management, and a changing role of informal caregivers, the demand for health care delivery, and the role of technology are changing rapidly. To contain health care costs and maintain the quality of care and living, governments direct policies to stimulate eHealth to increase and support self-management [3]. In this study, eHealth is defined in line with the description by Eysenbach [4] and the different taxonomies described by Oh et al [5]: eHealth is at the intersection of medical informatics, public health, and business and offers health services to support care delivery, manage care, promote prevention, and educate; it is delivered or enhanced through the internet and related technologies (eg, domotics, wearables, and sensors). In the domain of older persons living at home, we define eHealth as web-based contact possibilities between the clients and health care providers and applications for self-management, telehomecare, telemedicine, telemonitoring, and telerehabilitation.

eHealth has shown to be valuable in promoting medication adherence and improving self-management in the population of older persons [6]. In addition, eHealth can be used to monitor clinical signs, collect health information, support users in activities related to their health, and promote a healthy lifestyle or arrange remote consultations [7-10]. Growing internet access, increasing use of mobile apps, and current technology trends create opportunities for novel services and new forms of health care through eHealth [11,12]. Governments are also increasingly funding initiatives that replace traditional care with alternatives that use information and communication technologies to remotely monitor and deliver health care services. Primary funding motivation is economic in nature—promoting preventive measures to avoid costly consequences and stimulate efforts to increase access to care [13].

Although eHealth is frequently considered a promising development, these applications are not without considerable costs. eHealth equipment must be purchased, and systems must be operated and maintained. Data recorded by eHealth should be monitored. However, frequently, the stakeholder who benefits from the application is not the same stakeholder who is paying for it. Costs and benefits affect different stakeholders and potentially also at different points in time; therefore, the economic interests of stakeholders are often not aligned. From the health provider perspective, such an investment does not make an economic sense, whereas it might be highly valuable from a societal perspective, considering the total benefits and costs regardless of where they occur. Any assessment of the value of an eHealth application, therefore, requires considering multiple stakeholders in a holistic and integrated assessment of all costs (ie, the direct and indirect and short- and long-term costs) and all benefits (ie, the direct and indirect and short- and long-term *gains*) of an eHealth application. This rationale represents the core of economic evaluations in health care [14]. Economic evaluations in health care come in different forms such as cost-effectiveness analyses (CEAs), cost utility analyses (CUAs), and cost-benefit analyses (CBAs). Regardless of the type, they have in common considering both costs and benefits, that is, what we have to give up *and* what we will gain. The main difference is the way in which *gain* is incorporated: CEAs consider a one-dimensional measure of the *gain*, which only allows for a comparison of programs with the same effect measures. CUAs assess gain through utility, frequently in quality-adjusted life years, which is comparable between health programs. CBAs assess the gain monetarily, that is, costs and outcomes are directly on the same scale [14,15].

### Research Objective

If an interested stakeholder wants to conduct an economic evaluation, that is, apply such an evaluation to a specific case, he or she must first identify the relevant cost and benefit elements. For this identification step, the body of literature can serve as a valuable source of information. Currently, it is unclear what types of information are available in the literature about the relevant cost and benefit elements of eHealth. Therefore, we conducted a scoping review to systematically map the literature in this area and to develop an up-to-date framework for conducting all-inclusive economic evaluations of eHealth applications that support independent living of older persons.

## Methods

### Scoping Review

Our research methodology was drafted using the PRISMA (Preferred Reporting Items for Systematic Reviews and Meta-analysis) protocols, specifically for scoping reviews. Among other things, scoping reviews are appropriate for identifying key characteristics and factors related to a concept [16]. Consequently, they lend themselves naturally to our research objectives.

### Search Query and Inclusion and Exclusion Criteria

**Figure 1:**
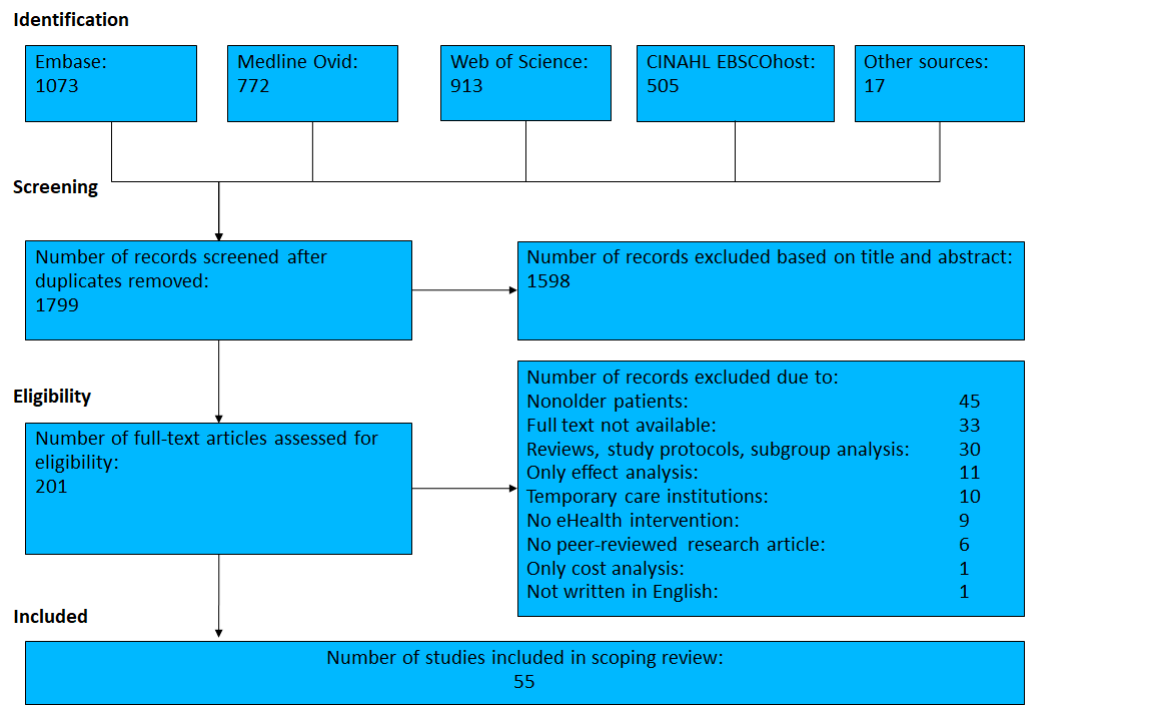
Flowchart of paper selection.

For this study, we identified papers published until December 2019 using the Embase, Medline Ovid, Web of Science, and CINAHL EBSCOhost databases. Additional papers were identified by scanning the references of the identified papers (indicated as other sources in the PRISMA flowchart in Figure 1). An experienced librarian developed search strings with some unique features to combine search terms effectively [17,18] and conducted the search. Our search terms are derived from the inclusion and exclusion criteria specified later, and the complete search query is provided in Multimedia Appendix 1.

The inclusion and exclusion criteria were specified to guide the identification process. We included original papers that were peer reviewed, had an empirical or prescriptive nature, and were written in English. Research protocols, commentaries, and editorial papers were excluded from the study. We also excluded reviews to avoid duplicate findings and avoid relying on the review’s interpretation of the cost and benefit labels. Concerning the patient population, we included papers that focused on patients with an average age of at least 65 years and who were living independently at their usual place of residence. Studies were excluded if they considered nonolder persons (ie, infants, adolescents, and a sample mean age younger than 65 years) or if the patient population received care in any institutionalized form (ie, hospitals, nursing homes, rehabilitation clinics, and hospice). In terms of the eHealth application, we focused on eHealth applications with web-based contact possibilities between clients and health care providers, including mobile health (mHealth) apps, and applications for self-management, telehomecare, telemedicine, telemonitoring, telerehabilitation, and active healthy aging technologies for older persons. Papers with the following technologies were excluded: papers describing technology not connected to the internet (eg, implantable cardioverter defibrillator) and papers on health information systems, electronic health records, robotics, and telephone consults only. To obtain a comprehensive overview, we did not use a strict definition of economic evaluation and did not constrain our search to particular types of economic evaluations. The only constraint on which we relied was that economic evaluations required both costs and benefits. Therefore, we included papers that measured at least one monetary aspect and one benefit of eHealth applications in any shape or form (eg, medical expenditures and analyzing clinical effects). Papers that only described or analyzed costs at an aggregated level or (clinical) effects were excluded.

### Screening and Eligibility

Initially, 2 of the authors (SS and HE) screened a small sample simultaneously to align the assessment. Subsequently, SS and HE conducted abstract and title screening, with each screening half of the identified references. The studies were labeled *not relevant* or *potentially relevant* based on the inclusion criteria. A random sample of the references was blindly double-checked by AW and RH. The random set has been created by selecting every 10th article from the set of annually ordered articles. Interrater reliability was evaluated using the Cohen kappa (κ) index, which is a robust statistic useful for interrater reliability testing [19]. This double screening yielded a *fair* overlap between the assessments by SS or HE and AW or RB (Cohen κ index=0.28). After realigning the inclusion and exclusion criteria and rescreening all of the references with previous disagreements (conducted by AW), substantial improvements in the alignment were achieved (Cohen κ index=0.75).

In the next screening round, SS and HE conducted the screening based on full texts, and both authors went independently through all of the references initially labeled as *potentially relevant*. This double-blind screening yielded a *substantial* overlap (Cohen κ index=0.64). SS and HE discussed all of the references for which their assessments diverged and thus resolved disagreements.

### Data Extraction and Categorization

Two authors (SS and HE) extracted the following information from the studies: patient population, country, type of eHealth intervention, type of analysis that the papers report performing, the specific cost types that the studies considered, and the specific benefits, gains, and consequences that the studies included. Subsequently, we categorized the costs and benefits. We started with 4 categories pertaining to (1) implementation activities for the eHealth application, (2) operating and maintenance activities for the eHealth application, (3) processes of health care delivery, and (4) outcomes. While extracting the information, we further refined these categories depending on activities, stakeholders, and institutions such that the subcategories were mutually exclusive but sufficiently broad to capture related items.

## Results

### Study Selection

The search and screening processes are shown in Figure 1. The database and manual searches resulted in 1799 papers. After the first round of removal of duplicate papers and title and abstract screening, the remaining 201 papers were subjected to full-text reading. After the second step, 55 papers met our inclusion criteria and were included in the final set for analysis. The included references were published in the 2000-2019 time frame, with 5 included papers from the first 5-year period (2000-2004), 12 between 2005 and 2009, 17 between 2010 and 2014, and 21 between 2015 and 2019.

### Patient Characteristics

The patient characteristics were rather broad; however, most studies focused on chronic or cardiovascular diseases. Of the 55 references, 13 had a patient population with various chronic health problems [13,20-31]. Cardiovascular problems received specific attention in 17 studies [32-48]. In 10 studies, the focus was on chronic obstructive pulmonary disease [49-58], and in 2 studies, the focus was on chronic skin problems [59,60]. Other studies considered diabetes [61], age-related macular degeneration [62], post-knee arthroplasty patients [63], Parkinson disease [64], and terminal patients [65]. Five studies did not specify any health conditions or diseases [66-70]. Of the 55 studies, 3 focused on mental and behavioral disorders such as anxiety [71], dementia [72], and depression [73].

### Country

The studies in English were geographically clustered, with 26 studies conducted in North America, among which 20 occurred in the United States [20-23,25-28,30,31,33,34,37,38,42,61, 62,64,67,68] and 6 in Canada [29,40,49,51,63,66]. Among the 22 studies conducted in Europe, they were spread across countries, with 5 studies conducted in England and the United Kingdom [24,41,43,44,53]; 3 each in Denmark [50,52,58], Italy [32,45,55], the Netherlands [35,39,72]; 2 each in Austria [46,60] and Germany [47,54]; and 1 each in France [59], Spain (although not explicitly stated in the paper) [57], Norway [70], and Sweden [69]. Finally, 4 studies took place in Australia [36,56,71,73] and 3 in Asia, among which 2 were in Japan [13,65] and 1 in Taiwan [48]. With the study in Taiwan being the only one conducted in a country that is not a part of the Organization for Economic Cooperation and Development, there was a strong emphasis on economically strong countries with aging populations.

### Type of eHealth Interventions

The majority of included studies focused on telemonitoring or remote monitoring involving the measurement of vital statistics and the transmission of patient data followed by an assessment—either automatically or manually—and triggering action by health care professionals if required [22-25,27-30,32-35,38-43,45-51,53,54,56,57,62,65,68,70]. In addition, other eHealth forms included in our review related to video consultations and virtual visits [20,21,23,30, 37,42,48,55,59,61,63,64,69]; deployment of sensor technology to analyze behavioral patterns and wireless transmitters [13,26,48,61,66,67,70,72]; email messaging services and web portal access [44,69,71,73]; online disease management courses or resources [44,71,73]; internet-delivered cognitive behavioral therapy [71,73]; remotely supervised rehabilitation activities, such as assistant mHealth [36,52,58]; and digital data transmission [60]. Note that some studies blended various eHealth forms and applications and that there was some ambiguity in the terminology, with telehealth often being used interchangeably with telemedicine, telemonitoring, or remote monitoring. The specific eHealth interventions analyzed in the studies are described in Multimedia Appendix 2 [13,20-73].

### Type of Analysis

The included studies indicated various types of analysis. In total, 18 studies reported CEAs [24,25,27,34,35,39-41,44,46, 50,53,54,59,60,70,71,73], 2 studies combined CEAs with clinical or budget impact analyses [48,62], 2 studies reported CUAs [36,52], and 1 study reported a CBA [13]; 8 studies denoted the evaluation as cost analysis [26,45,61,63,67-69,72], the term *cost minimization* was used in 4 studies [29,49,51,65], and 1 study reported relying on cost consequence analysis [43]. The remaining 17 studies stated that they evaluated a range of cost and outcome measures [21,23,28,30-33,37,38,42,47, 55-58,64,66], and 2 studies relied on case studies to outline benefits, saving, expenditures, and outcomes [20,22].

Of the 55 included studies, 8 relied on Markov modeling and simulation models with a time frame of 1 year [40,65], 5 years [13,34], 10 years [43,62], 20 years [35], up to 30 years [41]. Only a few studies without Markov modeling explicitly stated the time frame of the economic evaluation, with 1 study capturing a period of multiple weeks [63] and 6 studies with a 1-year time frame [24,39,44,45,50,54]. The remaining 40 studies did not indicate the time frame explicitly, and the period of data collection was, if applicable, used as a proxy for the time frame: 1-6 months [22,25,36-38,42,51,56,57,60,66], 7-12 months [28,29,48,52,58,59,64,67-69,71-73], 13-18 months [20,21,31, 53], 19-24 months [27,49,61], and beyond 24 months [26,30,32,33,46,47,55,70]. In 1 study, the period of data collection was insufficiently described [23].

### Specific Cost and Benefit Types

When we consider eHealth applications, what direct and indirect costs and benefits (or consequences) might be relevant to consider? Tables 1-3 provide an answer by outlining the different cost and benefit types that the studies included in our review considered. Table 1 shows the eHealth intervention costs categorized into implementation and operating activities. Table 2 provides an overview of the consequences of eHealth and focuses on resource usage. Finally, Table 3 depicts eHealth consequences in terms of outcomes categorized by stakeholder group.

**Table 1:** Intervention costs of eHealth applications.

Intervention costs		Considered by number of studies	References
**Implementation activities**			
	Device purchase (monitoring equipment, videoconferencing equipment, etc)	32	[13,22-25,29-31,35-37,39-41,44,47,50-53,55,56,59,61,63,65,66,69-73]
	License or software or initial fee purchase	7	[13,24,29,50,51,59,72]
	Equipment installation	18	[13,24,29,34,35,38,50-53,55,61,63,65,66,69,70,72]
	Training or education of operators	10	[24,25,29,35,44,50,52,53,61,70]
	Technician travel time to install equipment	5	[30,36,40,52,63]
**Operating activities**			
	Maintenance (server, host, call center, or station)	18	[13,24,26,28,29,35,37,39,40,43,50,51,53,55,59,65,66,68,69]
	Periodic fees (for licenses, insurance, etc)	15	[24,29,31,35,37,38,43,50,55,59,61,68,71-73]
	Medical staffing: reviewing or assessing or intervening	26	[13,24,29,32,34-36,38,40,41,44,50,53,55-57,61-63,65,66,69-73]
	Nonmedical staffing: technical support	14	[13,24,29,39,40,50,51,53,61,63,66,68,69,72]
	Technician travel time to maintain equipment	1	[24]

**Table 2:** Intervention consequences—resource usage.

Intervention consequences		Considered by the included studies, n	References
**eHealth usage by patient**			
	Televisits: number or duration of visits	17	[20,22,23,25,30-32,35,37,39,44,45,51,56,61,64,70]
**Health resource usage by patient—community health services or primary care**			
	Travel time or transportation costs (eg, for ambulance)	9	[39,44,46,53,55,59,60,64,67]
	General practitioner: number or duration of visits	16	[24,28,31,35,39,40,44,50,52,55-57,59,68,71,73]
	Walk-in center: number or duration of visits	2	[24,44]
	Physiotherapist: number of sessions	5	[24,39,52,59,63]
	Psychologist: number of sessions	2	[24,39]
	Community nurse: number or duration of visits	4	[24,39,44,50]
	Home care: number or duration of visits	27	[21-25,28-32,34,37-39,49-51,55-57,61,64-67,69,72]
	Meals on wheels	1	[24]
**Day services**			
	Day care	2	[24,69]
	Same-day surgeries	1	[67]
**Institutionalized care**			
	Rehabilitation clinics: number or duration of admissions	2	[50,54]
	Skilled nursing facilities: number or duration of admissions	6	[27,62,67,69,70,72]
	Long-term care: number or duration of admission	2	[24,67]
	Hospice: number or duration of admissions	2	[21,35]
**Hospital use**			
	Emergency department: number of visits	21	[21,24,27-29,31,33,35,37-39,42,49,50,52,53,55-57,67,68]
	Outpatient clinic: number or duration of visits to specialists	21	[21,24,27,28,34,35,40,43,45,46,48,50,52-54,56,59,60,62,67,68]
	Hospital: number or duration of admissions	40	[21,24,25,27-31,33,34,36-59,65-68,70,71,73]
	Intensive care unit: Admissions	2	[55,57]
**Drug treatment and laboratory diagnostics**			
	Medication, prescriptions, or medical supplies	16	[21,24,31,38,39,43,44,50,52,54,55,59,62,67,71,73]
	Laboratory	3	[66,67]

**Table 3:** Intervention consequences—stakeholder outcomes.

Intervention consequences		Considered by the included studies, n	References
**Patient or client outcomes**			
	Physical health status (mortality, morbidity, cardiovascular events, exacerbations, etc)	14	[30,32-34,43,44,46,47,54,55,59-61,68]
	Psychological health status (anxiety, depression, or empowerment)	4	[22-24,66]
	QALYs^a^	12	[24,35,36,39-41,44,50,52,62,71,73]
	Quality of life (if not measured in QALYs but differently)	11	[24,28,33,37,38,42,47,56,61,64,66]
	Setting-specific quality of care indicators	1	[31]
	Satisfaction (with the device or eHealth service)	8	[20,23,25,29,31,40,51,66]
	Satisfaction (in general)	5	[22,28,30,37,61]
	Patient experience or perceived benefits	3	[57,65,69]
	Well-being	1	[72]
	Time spent in the usual place of residence	1	[26]
	Transfer to a different level of care	1	[30]
	Time absent from work (productivity loss or loss of income)	2	[44,62]
	Device-related technical events	2	[43,47]
**Professional caregivers**			
	Satisfaction with the device	2	[20,25]
	Satisfaction in general	1	[30]
	Travel time to patient’s home	11	[20,25,28,30-32,49,51,56,63,65,70]
**Informal caregivers**			
	Time absent from work (productivity loss)	2	[62,70]
	Burden	1	[66]
	Well-being	1	[72]
**Transfer payments**			
	Attendance allowance (recipients receive payments to manage their own health)	1	[69]
	Respite care (payments made to relieve informal caregivers from providing care)	1	[69]

^a^QALYs: quality-adjusted life years.

### Level of Integration

**Figure 2:**
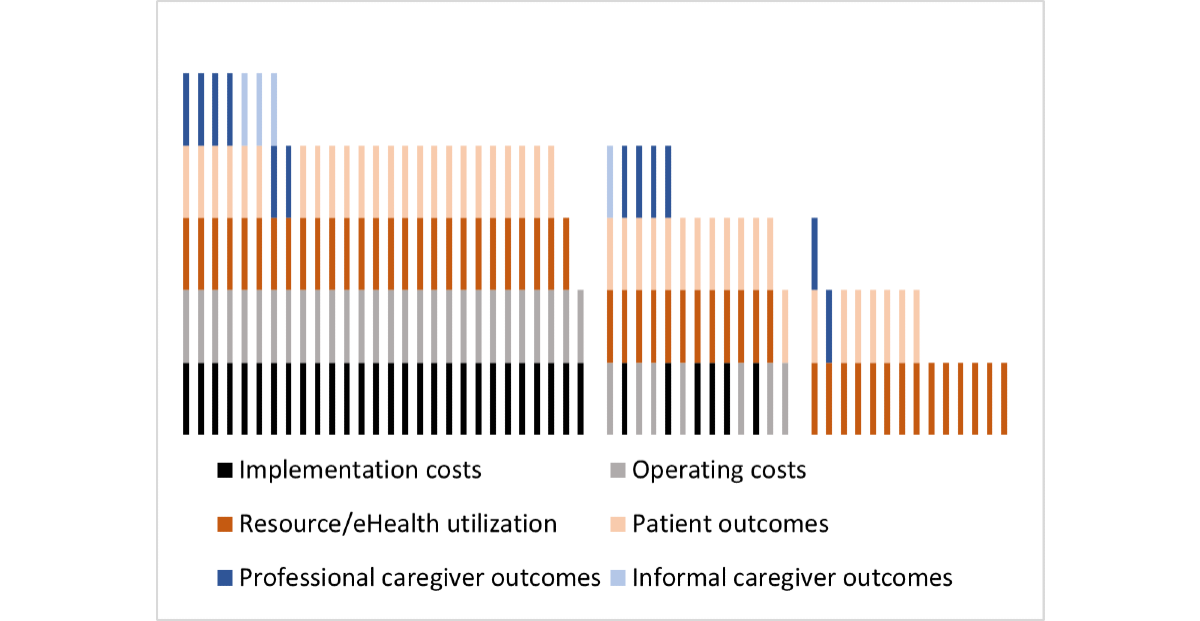
Level of integration in 55 included studies (represented as bars).

Although Tables 1-3 provide an extensive overview of cost and consequence aspects already considered in previous studies, they indicate that there is diversity in how many different aspects the studies have considered. For all the studies, we determined whether they included at least one element of implementation costs, operating costs, and consequences such as eHealth and resource usage, patient outcomes, professional caregiver outcomes, and informal caregiver outcomes. The studies had different foci and different levels of integration, as outlined by the diversity in Figure 2. Each study is represented by 1 bar decomposed into the cost and consequence subcategories considered in the study. The group of 28 studies on the left considered at least one implementation and one operating activity, in addition to the consequences of the eHealth intervention [13,24,29,31,34-41,44,50,51,53,55,56,59,61,63, 65,66,69-73]. The middle group of 13 studies contains either implementation or operating activities, in addition to consequences [22,23,25,26,28,30,32,43,47,52,57,62,68]. Finally, the 14 studies on the right do not consider implementation and operating activities but only focus on the consequences [20,21,27,33,45,46,48,49,54,58,60,64,67].

### Prominent and Lacking Features

Tables 1-3 also show that some components have received more attention than others. Concentrating on the different consequences, we observed the following: If patient outcomes are considered, the focus lies on physical outcomes [30,32-34,43,44,46,47,54,55,59-61,68] and quality of life or quality-adjusted life years [24,35,36,39-41,44,50,52,62,71,73]. Productivity loss because of time absent from work received less attention, with only 4% (2/55) of the studies incorporating it [44,62]; however, this outcome is understandable given that our review focused on the population of older persons, which predominantly no longer participates in the labor market. In terms of resource utilization, there is a strong emphasis on home care visits, indicated by 49% (27/55) of the included studies [21-25,28-32,34,37-39,49-51,55-57,61,64-67,69,72], and hospital usage covered, indicated by 73% (40/55) of the included studies [21,24,25,27-31,33,34,36-59,65-68,70,71,73]. This outcome can be explained by many of the included studies relying on remote monitoring or virtual visits to substitute for home care visits or to prevent exacerbations that lead to hospital admissions.

Comparing the consequences for professional caregivers and informal caregivers, it becomes obvious that certain elements are missing across all included studies. For instance, 4% (2/55) of the included studies considered the satisfaction of professional caregivers (in general or with the device) [20,25]; however, the satisfaction of informal caregivers was not captured in any of the included studies. Similarly, for professional caregivers, 20% (11/55) of the included studies captured the travel time to patients’ homes [20,25,28,30-32,49,51,56,63,65,70]; however, this aspect was neglected for informal caregivers. Conversely, for informal caregivers, emotional burden and well-being were captured by 4% (2/55) of the included studies [66,72], whereas none of the included studies focused on these 2 aspects for professional caregivers.

Our final observation relates to the research and development (R&D) costs of eHealth applications. In fact, none of the included studies considered R&D costs, which is understandable from the perspective that these costs have already been spent by the time that the eHealth application is set up and running. However, it implicitly assumes that R&D costs only occur before the eHealth intervention is set up and that there is no ongoing refinement.

## Discussion

### Principal Findings

We conducted this review to establish a framework of costs and benefits considered in the economic evaluations of eHealth applications that support the independent living of older persons. Our search identified 55 papers that conducted economic evaluations. All of the identified papers focused on independent living of older persons in their role as patients with one or more chronic conditions. The identified papers considered a range of different types of costs and benefits. Costs pertain to implementation activities and operating activities related to eHealth applications. Benefits (or consequences) can be categorized according to stakeholder groups, that is, patients, caregivers, and health care provider organizations. These benefits can be further divided into stakeholder-specific outcomes and resource utilization. Some cost and benefit types have received more attention than others. For instance, patient outcomes are predominantly captured via quality-of-life considerations and various types of physical health status indicators. From a resource utilization perspective, a strong emphasis is placed on home care visits and hospital usage. One reason for this emphasis is the frequency in which studies focus on remote monitoring to prevent unnecessary hospital admissions or to substitute for home care visits.

Our data extraction also revealed a set of elements that have not been considered across all of the identified papers, including travel time and satisfaction of informal caregivers, emotional burden and well-being of professional caregivers, and last but not least, the R&D costs of the eHealth application. The reasons why these aspects have been neglected can be manifold: they might have been irrelevant from the perspective from which the economic evaluation was carried out, they might have been unobservable because of long time lags beyond the time frame of the evaluation, or it might have been infeasible to capture and quantify these aspects because of methodological obstacles or data unavailability. That the initial R&D costs of the eHealth applications have not been considered can be justified with the sunk cost argument—by the time that the evaluation takes place, the R&D costs have already been spent. In this sense, the eHealth application is considered a static technology. In the longer run, however, the eHealth application might require an upgrade to comply with new laws and regulations, for reasons pertaining to data privacy, data security, data storage, or improvements in usability. Whether these upgrade costs are indeed relevant for future economic evaluations depends on the perspective and time frame.

Like any other technology, eHealth applications are subject to change. Fueled by the increasing availability of data, changes in law and regulations and improved usability, new fields, and areas of applications might emerge. Our framework is based on eHealth applications currently in place. Future eHealth applications might generate costs and benefits that are different from the eHealth applications on which our framework is based.

### Implications for Research and Practice

Economic evaluations of eHealth applications are gaining momentum, as indicated by the increasing number of publications and reviews [74,75]. Health economic frameworks and principles are described, and the steps to measure costs and benefits are emphasized [14,15]. Our review directly connects to the measurement aspect by focusing on its first step, that is, the identification of costs and benefits. We contribute to the body of literature by providing a detailed and up-to-date framework of cost and benefit categories.

If we consider eHealth, there are many stakeholders involved, such as patients, eHealth suppliers (formal and informal) caregivers, funding bodies, health authorities, and so on. Notably, these stakeholders are likely to attribute different values to eHealth applications because they are affected differently by their costs and benefits. This fact has consequences for investment and funding decisions, and it has long been argued that decision making remains hampered by the lack of reliable cost and benefit estimations [14,15]. To obtain reliable cost and benefit estimates, our framework could be of help by providing a starting point for the identification process. Our framework can support this goal; however, the measuring and valuing process must be context-specific and tailored to the case at hand. How to value components that, for instance, do not lend themselves naturally to being quantified in countable units, such as the feeling of safety [76], is worthy of study in itself and beyond the scope of our review. The variety that we observe in terms of costs and consequences considered in the economic evaluations and the type of analysis performed and the extent to which the costs and consequences are integrated indicates that the field has not yet reached consensus on a standard procedure for determining eHealth value. Whether and to what extent the observed variety is actually linked to the quality of the economic evaluation is an interesting avenue for future research.

Financial costs directed to patients or informal caregivers might be an obstacle to using the eHealth service or application or continuation of usage of these services, especially in countries where mandatory health insurance coverage is lacking. Although a recent study in the Netherlands identified finance as a factor not significantly related to intention to use medical apps among older persons [77], future studies should focus on how the costs are covered and who should pay for the direct or indirect costs of eHealth.

### Conclusions

A holistic and reliable value assessment of eHealth applications requires a profound understanding of the applications’ costs and benefits. The first step in measuring costs and benefits is identifying the relevant costs and benefit categories that the eHealth application affects. We conduct a scoping review to support this identification process by providing an overview of the direct and indirect costs and benefits that economic evaluations have incorporated so far. Our cost-and-benefit framework is particularly useful in the context of eHealth applications that support the independent living of older persons. As this patient group is expected to be increasingly targeted to contain health care costs, expanding the scope of eHealth applications to broader populations, rather than only diagnosed patient groups, and assessing the value of eHealth technologies are of the utmost importance for making well-informed investment and funding decisions.

## Appendix

Multimedia Appendix 1 Search strategy.

Multimedia Appendix 2 Type of eHealth intervention, patient population, country, and analysis type.

## Abbreviations

CBA: cost-benefit analysis
CEA: cost-effectiveness analysis
CUA: cost utility analysis
mHealth: mobile health
PRISMA: Preferred Reporting Items for Systematic Reviews and Meta-analysis
R&D: research and development
