# Supplementary material for: eHealth Applications to Support Independent Living of Older Persons: Scoping Review of Costs and Benefits Identified in Economic Evaluations
Source: J Med Internet Res. 2021 Mar 9;23(3):e24363. doi: 10.2196/24363 (PMC7988395; doi:10.2196/24363)
Supplement: Multimedia Appendix 1 [file jmir_v23i3e24363_app1.pdf]

APPENDIX 1

Search strategy

embase.com 1073

('economic evaluation'/exp OR 'health economics'/de OR 'program cost effectiveness'/de OR (((econom*) NEAR/3 (evaluat* OR analys* OR model*)) OR (cost NEAR/3 (eval* OR benefit* OR effectiv* OR utilit* OR consequen* OR minimi* OR outcome* OR reduc* OR saving* OR efficien*)) OR (value NEAR/3 money) OR (willingness NEAR/3 pay) OR (business NEAR/3 model*) OR (social NEAR/3 return NEAR/3 invest*)):ab,ti) AND ('teleHealth'/exp OR videoconferencing/exp OR telemetry/exp OR (e-coach* OR teleHealth OR telemonitor* OR Telerehabilitation* OR Telemedic* OR Telehomecare* OR telenursing OR telecard* OR telesurg* OR teledermato* OR Telepsychiatr* OR Teleconsult* OR Teleradiolog* OR Telecare* OR teleecho* OR Tele-toxico* OR Teletoxico* OR telepractice* OR tele-practice* OR telediagnos* OR Telehome-care* OR teleaudiolog* OR telepatholog* OR teleradiotherap* OR eHealth OR e-health OR mhealth OR m-health OR videoconferencing OR videocommunicat* OR ((mobile OR It) NEAR/3 (health OR solution*)) OR telesurveil* OR (remote NEAR/3 (monitor* OR sens*)) OR telemetr* OR ((tele* OR video OR e) NEXT/1 (psychiatr* OR consult* OR radiolog* OR health OR monitor* OR rehabilitation* OR medic* OR homecare* OR nursing OR card* OR surg* OR dermato* OR care* OR home-care* OR nursing OR echo* OR diagnos* OR audiolog* OR patholog* OR radiotherap* OR surveillan* OR conferencing OR communicat* OR metr* OR physiotherapy*)) OR Wearable*):ab,ti) AND ('home care'/exp OR 'independent living'/exp OR 'assisted living facility'/exp OR 'healthy aging'/exp OR 'community living'/de OR ('home care' OR homecare OR telehomecare OR home OR ((independent* OR assisted OR communit*) NEAR/3 (living)) OR ((healthy OR active) NEAR/3 (aging OR ageing)) OR domotic*):Ab,ti) NOT ([Conference Abstract]/lim) AND [English]/lim

Medline Ovid 772

(Cost-Benefit Analysis/ OR Economics, Medical/ OR (((econom*) ADJ3 (evaluat* OR analys* OR model*)) OR (cost ADJ3 (eval* OR benefit* OR effectiv* OR utilit* OR consequen* OR minimi* OR outcome* OR reduc* OR saving* OR efficien*)) OR (value ADJ3 money) OR (willingness ADJ3

pay) OR (business ADJ3 model*) OR (social ADJ3 return ADJ3 invest*)).ab,ti.) AND (exp Telemedicine/ OR Videoconferencing/ OR Telemetry/ OR (e-coach* OR teleHealth OR telemonitor* OR Telerehabilitation* OR Telemedic* OR Telehomecare* OR telenursing OR telecard* OR telesurg* OR teledermato* OR Telepsychiatr* OR Teleconsult* OR Teleradiolog* OR Telecare* OR teleecho* OR Tele-toxico* OR Teletoxico* OR telepractice* OR tele-practice* OR telediagnos* OR Telehome-care* OR teleaudiolog* OR telepatholog* OR teleradiotherap* OR eHealth OR e-health OR mhealth OR m-health OR videoconferencing OR videocommunicat* OR ((mobile OR It) ADJ3 (health OR solution*)) OR telesurveil* OR (remote ADJ3 (monitor* OR sens*)) OR telemetr* OR ((tele* OR video OR e) ADJ (psychiatr* OR consult* OR radiolog* OR health OR monitor* OR rehabilitation* OR medic* OR homecare* OR nursing OR card* OR surg* OR dermato* OR care* OR home-care* OR nursing OR echo* OR diagnos* OR audiolog* OR patholog* OR radiotherap* OR surveillan* OR conferencing OR communicat* OR metr* OR physiotherapy*)) OR Wearable*).ab,ti.) AND (exp Home Care Services/ OR Independent Living/ OR exp Assisted Living Facilities/ OR Healthy Aging/ OR (home care OR homecare OR telehomecare OR home OR ((independent* OR assisted OR communit*) ADJ3 (living)) OR ((healthy OR active) ADJ3 (aging OR ageing)) OR domotic*).ab,ti.) NOT (news OR congres* OR abstract* OR book* OR chapter* OR dissertation abstract*).pt. AND english.la.

CINAHL EBSCOhost 505

(MH Cost-Benefit Analysis OR TI (((econom*) N2 (evaluat* OR analys* OR model*)) OR (cost N2 (eval* OR benefit* OR effectiv* OR utilit* OR consequen* OR minimi* OR outcome* OR reduc* OR saving* OR efficien*)) OR (value N2 money) OR (willingness N2 pay) OR (business N2 model*) OR (social N2 return N2 invest*)) OR AB (((econom*) N2 (evaluat* OR analys* OR model*)) OR (cost N2 (eval* OR benefit* OR effectiv* OR utilit* OR consequen* OR minimi* OR outcome* OR reduc* OR saving* OR efficien*)) OR (value N2 money) OR (willingness N2 pay) OR (business N2 model*) OR (social N2 return N2 invest*))) AND (MH Telemedicine+ OR MH Videoconferencing OR MH Telemetry OR TI (e-coach* OR teleHealth OR telemonitor* OR Telerehabilitation* OR Telemedic* OR Telehomecare* OR telenursing OR telecard* OR telesurg* OR teledermato* OR Telepsychiatr* OR Teleconsult* OR Teleradiolog* OR Telecare* OR teleecho* OR Tele-toxico* OR Teletoxico* OR telepractice* OR tele-practice* OR telediagnos* OR Telehome-care* OR

teleaudiolog* OR telepatholog* OR teleradiotherap* OR eHealth OR e-health OR mhealth OR m-health OR videoconferencing OR videocommunicat* OR ((mobile OR It) N2 (health OR solution*)) OR telesurveil* OR (remote N2 (monitor* OR sens*)) OR telemetr* OR ((tele* OR video OR e) N1 (psychiatr* OR consult* OR radiolog* OR health OR monitor* OR rehabilitation* OR medic* OR homecare* OR nursing OR card* OR surg* OR dermato* OR care* OR home-care* OR nursing OR echo* OR diagnos* OR audiolog* OR patholog* OR radiotherap* OR surveillan* OR conferencing OR communicat* OR metr* OR physiotherapy*)) OR Wearable*) OR AB (e-coach* OR teleHealth OR telemonitor* OR Telerehabilitation* OR Telemedic* OR Telehomecare* OR telenursing OR telecard* OR telesurg* OR teledermato* OR Telepsychiatr* OR Teleconsult* OR Teleradiolog* OR Telecare* OR teleecho* OR Tele-toxico* OR Teletoxico* OR telepractice* OR tele-practice* OR telediagnos* OR Telehome-care* OR teleaudiolog* OR telepatholog* OR teleradiotherap* OR eHealth OR e-health OR mhealth OR m-health OR videoconferencing OR videocommunicat* OR ((mobile OR It) N2 (health OR solution*)) OR telesurveil* OR (remote N2 (monitor* OR sens*)) OR telemetr* OR ((tele* OR video OR e) N1 (psychiatr* OR consult* OR radiolog* OR health OR monitor* OR rehabilitation* OR medic* OR homecare* OR nursing OR card* OR surg* OR dermato* OR care* OR home-care* OR nursing OR echo* OR diagnos* OR audiolog* OR patholog* OR radiotherap* OR surveillan* OR conferencing OR communicat* OR metr* OR physiotherapy*)) OR Wearable*)) AND (MH Home Health Care+ OR MH Community Living OR MH Assisted Living+ OR MH Healthy Aging OR TI (home care OR homecare OR telehomecare OR home OR ((independent* OR assisted OR communit*) N2 (living)) OR ((healthy OR active) N2 (aging OR ageing)) OR domotic*) OR AB (home care OR homecare OR telehomecare OR home OR ((independent* OR assisted OR communit*) N2 (living)) OR ((healthy OR active) N2 (aging OR ageing)) OR domotic*)) NOT PT (news OR congres* OR abstract* OR book* OR chapter* OR dissertation abstract*).pt. AND LA(English))

Web of science 913

TS=(((((econom*) NEAR/2 (evaluat* OR analys* OR model*)) OR (cost NEAR/2 (eval* OR benefit* OR effectiv* OR utilit* OR consequen* OR minimi* OR outcome* OR reduc* OR saving* OR efficien*)) OR (value NEAR/2 money) OR (willingness NEAR/2 pay) OR (business NEAR/2 model*) OR (social NEAR/2 return NEAR/2 invest*))) AND ((e-coach* OR teleHealth OR telemonitor* OR

Telerehabilitation* OR Telemedic* OR Telehomecare* OR telenursing OR telecard* OR telesurg* OR teledermato* OR Telepsychiatr* OR Teleconsult* OR Teleradiolog* OR Telecare* OR teleecho* OR Tele-toxico* OR Teletoxico* OR telepractice* OR tele-practice* OR telediagnos* OR Telehome-care* OR teleaudiolog* OR telepatholog* OR teleradiotherap* OR eHealth OR e-health OR mhealth OR m-health OR videoconferencing OR videocommunicat* OR ((mobile OR It) NEAR/2 (health OR solution*)) OR telesurveil* OR (remote NEAR/2 (monitor* OR sens*)) OR telemetr* OR ((tele* OR video OR e) NEAR/1 (psychiatr* OR consult* OR radiolog* OR health OR monitor* OR rehabilitation* OR medic* OR homecare* OR nursing OR card* OR surg* OR dermato* OR care* OR home-care* OR nursing OR echo* OR diagnos* OR audiolog* OR patholog* OR radiotherap* OR surveillan* OR conferencing OR communicat* OR metr* OR physiotherapy*)) OR Wearable*)) AND (("home care" OR homecare OR telehomecare OR home OR ((independent* OR assisted OR communit*) NEAR/2 (living)) OR ((healthy OR active) NEAR/2 (aging OR ageing)) OR domotic*))) AND DT=(article) AND LA=(english)
