# Supplementary material for: eHealth Applications to Support Independent Living of Older Persons: Scoping Review of Costs and Benefits Identified in Economic Evaluations
Source: J Med Internet Res. 2021 Mar 9;23(3):e24363. doi: 10.2196/24363 (PMC7988395; doi:10.2196/24363)
Supplement: Multimedia Appendix 2 [file jmir_v23i3e24363_app2.pdf]

APPENDIX 2

Type of eHealth intervention, patient population, country, and analysis type

Numeric Reference	Publication year	eHealth intervention	Patient population	Country where study was executed	Type of analysis paper reports to have conducted
[13]	2017	Telehomecare service with health monitoring using biometric sensor technology.	Variety of (chronic) health problems/ chronic diseases	Japan	CBA
[20]	2000	The telemedicine model used in this study is teleconsulting via video.	Variety of (chronic) health problems/ chronic diseases	USA	Case study of benefits and savings.
[21]	2017	Telepalliative care: virtual visits with members of the team, support for nurses from remote physicians during the visits.	Variety of (chronic) health problems/ chronic diseases	USA	Impact evaluation on cost and resource utilization.
[22]	2005	Telehealth monitoring: measuring vital statistics that can be assessed in the next telehealth (video-conferencing) encounter.	Variety of (chronic) health problems/ chronic diseases	USA	Patient selection, costs, projected savings, patient satisfaction, and the technical, clinical, and patient problems with the telehealth

					system are discussed.
[23]	2005	Telehealth system consisting of a patient unit and clinician unit. Monitoring equipment that measures patient data and transmits it to the nursing station.	Variety of (chronic) health problems/ chronic diseases	USA	Case study of expenditures and outcomes.
[24]	2013	Telehealth = Telemonitoring (transmission of data on vital signs for realtime monitoring) and telephone support.	Variety of (chronic) health problems/ chronic diseases	England	CEA
[25]	2009	Telemonitoring, transmission of physiological patient data and assessment by nurse.	Variety of (chronic) health problems/ chronic diseases	USA	CEA
[26]	2015	Sensor technology at independent housing facility, in addition to usual care. Sensors assist health status trend monitoring with non-wearables.	Variety of (chronic) health problems/ chronic diseases	USA	Cost analysis
[27]	2009	Assistive technology/adaptive equipment in the patients' homes. Patients' vital signs were remotely monitored.	Variety of (chronic) health problems/ chronic diseases	USA	CEA
[28]	2004	Home telehealth plus nurse case management. Peripheral devices monitored vital signs and out -of-range data triggered electronic alerts.	Variety of (chronic) health problems/	USA	Compare outcomes for subjective and objective quality-

			chronic diseases		of-life measures, health resource use, and costs.
[29]	2013	Home telemonitoring is the remote transmission (by patients) and collection (by healthcare professionals) of physiological and biological data (such as vital signs and symptoms) and behavioral data (such as compliance with medication and activity level) for the purposes of patient monitoring, data interpretation and clinical decision making.	Variety of (chronic) health problems/ chronic diseases	Canada	Cost-minimization analysis
[30]	2006	Home telehealth, two arms: Video group: standard home healthcare plus two virtual visits and internet access. Monitoring group: the same as video group plus home-based physiologic monitoring.	Variety of (chronic) health problems/ chronic diseases	USA	Evaluation patient outcomes, costs, and satisfaction.
[31]	2000	Remote video technology (video visits in addition to in-person and telephone visits)	Variety of (chronic) health problems/ chronic diseases	USA	Compare outcomes and cost savings
[32]	2013	Remote pacemaker in home control service run by nurses with data automatically transmitted.	Cardiovascular problems	Italy	Comparison of applicability, efficacy, and cost.
[33]	2014	Telemonitoring, transmission of physiological patient data and assessment by nurse practitioner.	Cardiovascular problems	USA	Effect identification on medical costs, 30-day rehospitalization, mortality, and

					health-related quality of life.
[34]	2016	Telehealth involving in-house monitoring and detection of deterioration and early treatment and intervention upon detection.	Cardiovascular problems	USA	CEA
[35]	2018	Home telemonitoring, patient data monitored daily and nurse telephone support.	Cardiovascular problems	Netherlands	CEA
[36]	2018	Telerehabilitation program: a 12-week exercise and education intervention. Telerehabilitation is the delivery of rehabiliation services at distance using information and communication technology. Exercise programmes were supervised remotely.	Cardiovascular problems	Australia	CUA
[37]	2001	Telecare: virtual home visits by nurses (video technology).	Cardiovascular problems	USA	Comparison of effectiveness & cost analysis.
[38]	2006	Home telemonitoring (remote monitoring) after hospital discharge. Patients transmitted monitor readings that were assessed by a nurse.	Cardiovascular problems	USA	Outcome comparison
[39]	2013	Home telemonitoring. Patients vital signs transmitted daily to categorize risk profile of patients and potentially triggering action by the nurse.	Cardiovascular problems	Netherlands	CEA
[40]	2013	Telehealth: remote delivery or monitoring where healthcare professional deliver services through information and communication technology. Condition	Cardiovascular problems	Canada	CEA

		management via nursing call center and home monitoring strategy.			
[41]	2013	Telemonitoring: physiological data is electronically transmitted to a healthcare team.	Cardiovascular problems	UK	CEA
[42]	2019	Home telemonitoring including weekly virtual nursing visits and daily monitoring and transmission of symptoms and vital signs.	Cardiovascular problems	USA	Outcome comparison between groups relying on an intention-to-treat analysis.
[43]	2013	Remote monitoring with clinical and technical data transmitted automatically, instant automated alert transmission in case of a prespecified parameter deviation.	Cardiovascular problems	UK	Cost-consequence analysis
[44]	2016	Online web portal access, online disease management resources.	Cardiovascular problems	England	CEA
[45]	2017	In-house monitoring. Data collected by the device is transmitted to a central database.	Cardiovascular problems	Italy	Cost analysis
[46]	2013	Home monitoring: mobile transmitter or cardiac messenger. The devices transmit clinical relevant data to the medical center allowing for remote monitoring.	Cardiovascular problems	Austria	CEA
[47]	2017	Home monitoring: autonomously data transmission from the pacemaker to central service center where the data is evaluated automatically and triggers sent to physicians.	Cardiovascular problems	Germany	Examining safety and efficacy/ effectiveness.
[48]	2013	Biometric sensor technology, televisits, telesurveillance. Real-time transition of biometrics from the patient to the	Cardiovascular problems	Taiwan	CEA & clinical impact analysis.

		healthcare team. Health records were under surveillance by the Telehealth center.			
[49]	2013	Telemonitoring/remote transition of patient data for patient monitoring and assisting in clinical decision making.	COPD	Canada	Cost-minimization analysis
[50]	2017	Telehealthcare equipment and monitoring by a municipality-based healthcare team. Patient data transmitted electronically and assessed by healthcare professionals.	COPD	Denmark	CEA
[51]	2006	Telehomecare, also called telemonitoring, remotely monitoring the patient´s health status with information and communication technology (Webphone with an integrated touch screen and modem). Nurse reviewed the data and intervened if necessary.	COPD	Canada	Cost-minimization analysis
[52]	2012	Telerehabilitation; preventive home monitoring concept through self-monitoring, aiding patients to maintain rehabilitation activities in their own home. Using telehomecare technology that involves transmission of patient data and assessed by healthcare professionals.	COPD	Denmark	CUA
[53]	2018	In-house monitoring involving data transmission and assessment by nurses.	COPD	England	CEA
[54]	2017	Home telemonitoring: vital parameters automatically transmitted, automatic risk assessment and follow up by professionals in case of high exacerbation probability.	COPD	Germany	CEA
[55]	2009	Tele-assistance programme, possibility of real-time tele-consultation.	COPD	Italy	Comparison of end-points.

[56]	2013	Telemonitoring, transmission of data, assessment by nurse.	COPD	Australia	Outcome comparison
[57]	2018	Telemonitoring for patients with early discharge from the hospital (home hospitalization). Transmission of physiological patient data and assessment by nurse.	COPD	Spain	Evaluation of usefulness & result comparison.
[58]	2012	Telerehabilitation: Preventive self-monitoring using a telehealth monitor, data transmission and assessment by healthcare professionals.	COPD	Denmark	Comparison of costs and hospital admissions.
[59]	2018	Telemedicine for follow-up, used for remote consultations to examine, monitor, and care for the patients at home.	Chronic skin problems (chronic wounds/chronic leg ulcers)	France	CEA
[60]	2007	Transmission of digital images to wound care center.	Chronic skin problems (chronic wounds/chronic leg ulcers)	Austria	CEA
[61]	2001	Telehomecare (video visits in addition to skilled nursing visits), biometric sensor technology.	Diabetics	USA	Cost analysis
[62]	2017	Home telemonitoring: home-based daily visual field monitoring system to detect changes in vision and triggering alerts.	Age-related macular degeneration	USA	CEA & societal net cost analysis & budget impact analysis.
[63]	2015	Videoconferencing in telerehabilitation.	Post-knee arthroplasty patients	Canada	Cost analysis

[64]	2013	Virtual visits (web-based video conferencing) by specialty care.	Parkinson	USA	Evaluate feasibility, effectiveness, and economic benefits.
[65]	2006	Home-based telepalliative care: Recording of vital signs and telecommunication between patient and palliative team.	Terminal patients	Japan	Cost-minimization analysis
[66]	2006	Telesurveillance: Telephone and wireless emergency call transmitter.	No specific condition	Canada	Evaluate the effectiveness and costs.
[67]	2017	Fixed and wearable sensors (ambient assisted living technologies).	No specific condition	USA	Cost analysis
[68]	2015	Telemonitoring, transmission of physiological patient data and assessment by nurse practitioner and intervention if needed.	No specific condition	USA	Cost analysis
[69]	2005	Bundled intervention consisting of: multimedia caring programmes, videophone access to professionals, internet access and email services.	No specific condition	Sweden	Cost analysis
[70]	2011	Devices such as fall sensors, global positioning system, and smoke detectors to enable older persons to live longer in their homes. The devices are connected to a personal computer at the home care providers' offices and then further to the on-call nurse's mobile phone. Each time unusual scores on the devices arise in the home of a patient, the phone alarms the nurse. Second intervention: Video visits imply that the nurse calls the patient via the television.	No specific condition	Norway	CEA

[71]	2015	Online internet-delivered cognitive behavioural therapy program with regular support from a clinical psychologist via secure email system and telephone.	Anxiety	Australia	CEA
[72]	2013	Assistive/sensor technologies. Equipment and sensors installed at home to register patient's behaviour. Information is sent to a server which can be accessed by a formal caregiver to assist in clinical decision-making.	Dementia	Netherlands	Cost analysis
[73]	2015	Online disease-management course "Managing your Mood", internet-delivered cognitive behavioural therapy, also including email messaging facilities with a therapist.	Depression	Australia	CEA
